# Quality of life in hemodialysis diabetic patients: a multicenter cross-sectional study from Palestine

**DOI:** 10.1186/s12882-018-0849-x

**Published:** 2018-02-28

**Authors:** Sohaib T. Khatib, Mohammad K. Hemadneh, Samer A. Hasan, Emad Khazneh, Sa’ed H. Zyoud

**Affiliations:** 1 Department of Medicine, College of Medicine and Health Sciences, An-Najah National University, Nablus, 44839 Palestine; 2 Nephrology Unit, Internal Medicine Department, An-Najah National University Hospital, An-Najah National University, Nablus, 44839 Palestine; 3 Poison Control and Drug Information Center (PCDIC), College of Medicine and Health Sciences, An-Najah National University, Nablus, 44839 Palestine; 4 Department of Clinical and Community Pharmacy, College of Medicine and Health Sciences, An-Najah National University, Nablus, 44839 Palestine

**Keywords:** Diabetes mellitus, Hemodialysis, Health-related quality of life, Palestine, HRQOL

## Abstract

**Background:**

Both diabetes and hemodialysis can seriously impair patients’ health related quality of life (HRQOL). This study seeks to obtain data which will help to address the factors associated with impaired HRQOL in hemodialysis patients with diabetes in Palestine.

**Methods:**

A cross-sectional study was performed in multiple centers in the period from November 2016 to June 2017. We utilized the Arabic version of EuroQoL 5 Dimensions 5 Levels (EQ-5D-5L) scale and EuroQol-visual analogue scale (EQ-VAS) to measure patients’ HRQOL. The study was conducted in six dialysis centers in the North of West Bank, Palestine. Descriptive and comparative statistics were used to describe clinical and socio-demographic features of patients. Multiple linear regression analysis was used to determine the association between clinical and socio-demographic factors and HRQOL score.

**Results:**

One hundred and forty one diabetic patients undergoing hemodialysis were enrolled in our study. Overall, 52.5% of them (74 patients) were males; the patients had a mean age of 60.32 with 52.5% of them aged below 60. The mean ± standard deviation of EQ-5D-5L index and EQ-VAS score was 0.314 ± 0.4 and 50.85±22.43, respectively. The findings of this study suggest that female patients, uneducated patients, unemployed patients, unmarried patients, and patients with more chronic diseases and comorbidities had a significant poor HRQOL scores (*p* values <0.05). Variables such as marital status and occupational status were significantly (*p* < 0.05) associated with the QOL score. More specifically, married status and employed patients positively associated with QOL score (β = 0.22; p = 0.016 and β = 0.27; p = 0.013, respectively).

**Conclusions:**

Among diabetic patients undergoing hemodialysis, married status and being employed were associated with modestly higher scores of QOL. We recommend that healthcare providers give more attention to diabetic dialysis patients who are unemployed and unmarried, as they are at a higher risk of having impaired HRQOL.

**Electronic supplementary material:**

The online version of this article (10.1186/s12882-018-0849-x) contains supplementary material, which is available to authorized users.

## Background

Type II diabetes mellitus (DM) is a major long-standing metabolic disorder causing a remarkable load on patients and their community in terms of mortality and morbidity [1]. It causes serious short-term and long-term complications; in the long term, it can lead to microvascular complications (e.g. nephropathy) and macrovascular complications (e.g. myocardial infarction) [2]. Diabetic nephropathy, which occurs in about one third of type 2 diabetic patients, is the most common factor leading to end-stage renal disease (ESRD). Despite recent therapeutic advances [3], its yearly incidence has more than doubled in the last decade to reach 44% of all ESRD [4]. The substantial increase in hemodialysis patients with DM is prone to rise even more as the population is getting older, the incidence of obesity is rising, and more people are surviving their cardiovascular incidents [5].

Health-related quality of life (HRQOL) is the individual’s beliefs, experiences, perceptions and expectations standing for enjoyment in those aspects of life probably influenced by health condition[6]. HRQOL is identified as a vital health outcome for studies evaluating the quality of healthcare, assessing the influence of illness and analyses of cost-effectiveness [7, 8]. Lower HRQOL scores lead to significant risk of hospitalization and mortality. HRQOL is a significant matter for dialysis patients and also for DM patients [9, 10]. The degree of HRQOL is considerably lower for those patients than for the public in spite of breakthroughs in the treatment of end-stage renal disease and DM [11]. Furthermore, HRQOL has been discovered to be a forecaster of mortality in both diabetic patients [12] and patients on hemodialysis [13, 14].

In reviewing the literature, there were several studies directed towards hemodialysis patients with DM. For example, Martínez-Castelao et al. [15] found that diabetic patients starting dialysis in Spain have lower HRQOL in contrast to non-diabetics. Another study showed that DM was one of the major determinants of mortality in patients on hemodialysis [5]. In Denmark, one study found that diabetic dialysis patients are characterized by reduced self-rated physical health, a high prevalence of diabetic complications, but relatively good mental health. Despite these studies, no one has specifically targeted the factors associated with impaired HRQOL among diabetic patients undergoing hemodialysis.

According to the 2016 annual health record of the Palestinian Ministry of Health, 5,761 new case of DM had been reported this year in West Bank [16]. Moreover, the total number of diabetic patients with nephropathy complications was 5,176. The overall number of dialysis patients in the West Bank has jumped from 687 patients in 2014 to 1,004 patients in 2015 which showed substantial increase in patients requiring hemodialysis [16].

Although HRQOL is a new research field in Palestine, multiple studies have recently examined HRQOL in Palestine among hypertensive [17], cancer [18], diabetic [6] and dialysis patients [19]. Despite these valuable studies, no one has specifically studied HRQOL among diabetic patients undergoing hemodialysis. Therefore, we carried out the study reported here to determine the patterns of HRQOL and to describe the significant factors associated with low HRQOL scores among Palestinian diabetic patients whose declining kidney function mandated initiation of renal replacement therapy using hemodialysis.

## Methods

### Study design

We performed this multicenter cross-sectional study in the period between November 2016 and August 2017.

### Study setting

The study was conducted in six dialysis centers in the North of West Bank, Palestine. These included the dialysis centers in Nablus, Tulkarem, Jenin, Qalqelia, Tubas and Salfeet. Five of them are centers at the government hospital in each city; only the center at Nablus is at a teaching hospital. We used annual health records of the Palestinian Ministry of Health to gather information regarding the numbers and distribution of diabetic and dialysis patients [16].

### Study population

At the time of our study, the total number of dialysis patients in these six cities was 507 patients; furthermore, the total number of hemodialysis machines was 92 machines. The distribution of patients and hemodialysis machines for each city was as follows: Nablus 210 patients (33 machines), Tulkarem 78 patients (13 machines), Jenin 113 patients (16 machines), Qalqelia 47 patients (10 machines), Tubas 27 patients (12 machines), Salfeet 32 patients (8 machines). Zyoud et al [19] found that 45% of hemodialysis patients in Palestine are diabetic, so the population of our study was estimated to be 230 patients.

### Sample size

We used a web-based calculator (Raosoft sample size calculator) to calculate our sample size, which was 141 patients. We increased our sample size by 7% to cover patients not responding to our study.

### Participants and sampling technique

One hundred and forty one patients were recruited by a reliable quota sampling method proportional to the number of patients in each dialysis center. We set five inclusion criteria for patients participating in our study, which were: (1) patients who have confirmed diagnosis of end-stage renal disease according to their medical files; (2) Patients who reported having type 2 DM; (3) Patients aged 18 years or more; (4) Patients who have been on regular hemodialysis for more than six months; (5) Patients with a history of DM for more than one year. We excluded patients with cognitive restrictions that made independent responses to the questionnaire impossible.

### Data sources and variables

The questionnaire we used to gather data from patients contained two sections, one for clinical history and socio-demographic information and the other for the validated Arabic version of HRQOL [20]. In the first section, we included the following socio-demographic characteristics: patient’s gender, age, weight and height (for calculating body mass index (BMI)), residency (village, city or Palestinian refugee camp) [6], educational level, living arrangements (living alone or with family), marital status, occupation, monthly household income, and whether the patient was a smoker or not. The clinical factors included in our study were: total number of chronic medications, total number of chronic diseases, time since DM was diagnosis, average duration of dialysis session, months on dialysis, history of kidney transplantation, and glycated haemoglobin (HbA1c) level. We categorized monthly household income as low (less than 400 Jordanian dinner (JD)), moderate (400–1000 JD) or high (more than 1000 JD) [17]. We also categorized HbA1c level as controlled (HbA1c ≤ 7%) or uncontrolled (HbA1c more than 7%) [21, 22]. We used the 5-level EuroQoL Group’s 5-dimension (EQ-5D-5L) questionnaire to estimate the health status and quality of life (QOL) for diabetic dialysis patients. EQ-5D is a standardized instrument for use as a measure of health outcome [23]. This questionnaire is a five-item scale assessing five separate dimensions of health: (MOBILITY, SELF-CARE, USUAL ACTIVITIES, PAIN/DISCOMFORT and ANXIETY/DEPRESSION). Each dimension has to be answered on a five–level scale (no problems, slight problems, moderate problems, severe problems and extreme problems). We also used the EQ visual analogue scale (EQ-VAS), which measures the subjects' perspectives on their QOL using a 100-point scale [23]. We used the Arabic version of EQ-5D [17, 19, 24] according to Euro QOL guidelines. We also registered our study with Euro quality of life and obtained permission for its use (ID: 20964). An additional file was supplied to illustrate detailed description of the study both in English and Arabic (Additional file 1). Cronbachs’ alpha for our study indicated acceptable internal consistency for EQ-5D-5L scale (α= 0.79). EQ-5D was scored using United Kingdom general population scoring algorithm (i.e. EQ-5D-5L Crosswalk Index Value Calculator) to calculate the index value using the value sets (weights) [25].

### Data collection procedure

After obtaining permission from the education department at Ministry of Health (MOH), we got access to patients’ medical records at each dialysis center to determine diabetic patients among those on hemodialysis. We also identified patients who met our inclusion criteria and invited them to participate in the study. We interviewed one hundred and forty one patients, we chose to meet them face-to-face to make the interview very comfortable for both patients and interviewers, and also to allow patients who aren’t able to read to have the chance to participate in our study. Interviews were carried out by qualified medical students from An Najah National University. After interviewing each patient, a blood sample was withdrawn from each patient by a qualified registered nurse at each dialysis center and these samples were sent to the laboratory department at An Najah National University Hospital to test the patients’ HbA1c level.

### Ethical approval

The Institutional Review Board (IRB) at An-Najah National University and Palestinian MOH approved our study protocol. We also obtained verbal consent from each patient before including him/her in our study. Approval for blood sample withdrawal was obtained from the MOH and from each patient.

### Statistical analysis

We entered our data and analyzed it using SPSS (IBM SPSS Statistic, version 21: IBM, Armonk, NY, USA) program. Data were expressed as mean (standard deviation) or median (interquartile range) or frequency (percentage). HRQOL was the dependent variable. Socio-demographic and hemodialysis related clinical factors were the independent factors. We used the Kruskal-Wallis or Mann-Whitney *U* test for data that were not normally distributed. We also assessed the normality of distribution of data using the Kolmogorov-Smirnov test. Median (interquartile range) was used to describe non-normally distributed variables. Internal consistency was assessed using Cronbach's α. The significance level was set at *P* < 0.05. We entered the independent variables such as gender, marital status, education level, occupational status, and total chronic diseases as dummy coding of 0 and 1. We tested associations between clinical and socio-demographic factors and HRQOL score with multiple linear regression to control for potential confounding factors.

## Results

### Socio-demographic and clinical features

A total of 141 diabetic patients undergoing hemodialysis participated in this cross sectional study out of 150 recruited, giving a response rate of 94%. Overall, 52.5% of them (74 patients) were males while 47.5% (67 patients) were females. Studied patients had a mean age of 60.32 with 52.5% of them aged below 60.The majority of our patients (44%) were obese with BMI above 30. Most of the recruited sample (81 patients) were living in a village, and 95.7% of patients in our sample were living with their family. The sample had a poor educational level, as only 28 (19.9%) graduated from school. The vast majority of patients were unemployed (89.4%), and so 71 out of 141 patients reported having low household income monthly. Regarding dialysis sessions, 87 patients (61.7%) had been having regular hemodialysis for less than four years and 123 patients (87.2%) were having 3 or more dialysis sessions weekly. Only 3 patients reported having a history of kidney transplant. 22.7% of patients were smokers and only 40 (28.4%) had three or more chronic diseases. Ninety-two patients (65.2%) were taking fewer than eight chronic medications. Other clinical and socio-demographic features are shown in Table 1.

**Table 1:** Socio-demographic and clinical features of the studied sample

Variable	Number (%) *n* = 141
Age category	
Age <60	74 (52.5)
Age >60	67 (47.5)
Gender	
Male	74 (52.5)
Female	67 (47.5 )
BMI	
Underweight	1 (0.7)
Normal range	37 (26.2)
Preobese (overweight)	41 (29.1)
Obese	62 (44)
Residency	
Village	81 (57.4)
City	47 (33.3)
Palestinian refugee camp	13 (9.2)
Living status	
Live with family	135 (95.7)
Live alone	6 (4.3)
Education level	
No formal education	17 (12.1)
Primary	35 (24.8)
Secondary	61 (43.3)
Graduated	28 (19.9)
Marital status	
Single , Widow ,Divorced	28 (19.9)
Married	113 (80.1)
Occupation	
Unemployed	126 (89.4)
Employed	15 (10.6)
Household income (month)	
Low (less than 400 JD ^a^)	71 (50.4)
Moderate (400–1000 JD)	57 (40.4)
High (more than 1000 JD)	13 (9.2)
Smoking	
Not smoker	109 (77.3)
Smoker	32 (22.7)
Diabetes duration	
1-3 years	8 (5.7)
4-5 years	3 (2.1)
More than 5 years	130 (92.2)
Dialysis vintage (Years)	
Less than 4 years	87 (61.7)
4 years or more	54 (38.3)
Dialysis frequency	
2 days or less	18 (12.8)
3 days or more	123 (87.2)
Dialysis session duration (hours)	
Less than 4 hours	120 (85.1)
4 hours or more	21 (14.9)
Kidney transplant	
Yes	3 (2.1)
No	138 (97.9)
Total chronic diseases	
None	7 (5)
One	48 (34)
Two	46 (32.6)
Three or more	40 (28.4)
Total chronic medications	
Less than 8	92 (65.2)
8 or more	49 (34.8)
HbA1c	
Controlled(≤7)	92 (65.2)
Uncontrolled(>7)	49 (34.8)

*Abbreviations*: *BMI* body mass index, *JD* Jordanian Dinar, *HbA1c* glycated haemoglobin

^a^1 Jordanian Dinar (JD) equals 1.41 US Dollar

### Quality of life and health status

**Fig 1:**
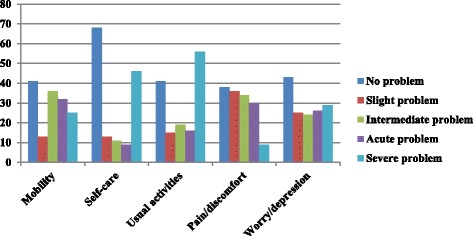
Distribution of HRQOL measures in different EQ-5D items among diabetic patients undergoing hemodialysis

In Palestinian diabetic patients undergoing hemodialysis in the North West Bank, we found that the mean EQ-5D-5L index value was 0.314, with median of 0.31 ± 0.4. The mean (± standard deviation) EQ-VAS score was 50.85 (±22.43). The number (percentile) of patients who answered “severe problems” for the five items of QOL was as follows: mobility 25 (17), usual activities 56 (38.1), self-care 46 (31.3), pain/discomfort 9 (6.1) and anxiety/depression 29(19.7) Fig. 1.

### EQ-5D-5L index values

The variables that displayed a statistically significant association with the EQ-5D were: patient’s gender, education level, marital status, occupational status, and total chronic diseases (all *P* values were less than 0.05). On the other side, patient’s age, BMI, residency, living status, household income, smoking, dialysis time, DM time, dialysis frequency, dialysis duration, kidney transplant, total chronic medications, and HbA1c level were not significantly associated with HRQOL (all *P* values were more than 0.05). For more details regarding the association between clinical and socio-demographic variables and EQ-5D total score, see Table 2.

**Table 2:** The association between EQ-5D total score and socio-demographic and clinical variables among diabetic patients undergoing hemodialysis (*n* = 141)

Variable	Number (%) *n* = 141	Median [1^st^ percentile-3^rd^percentile]	*P* value ^b^
Age category			
Age <60	74 (52.5)	0.36 [0.13-0.71]	0.130
Age >60	67 (47.5)	0.22 [-0.04-0.62]	
Gender			
Male	74 (52.5)	0.47 [0.13-0.76]	**0.001**
Female	67 (47.5 )	0.22 [-0.04-0.41]	
BMI			
Underweight	1 (0.7)		0.525
Normal weight	37 (26.2)	0.32 [0.1-0.7]	
Preobese (overweight)	41 (29.1)	0.34 [0.04-0.66]	
Obese	62 (44)	0.23 [-0.08-0.63]	
Residency			
Village	81 (57.4)	0.31 [-0.02-0.61]	0.645
City	47 (33.3)	0.26 [-0.07-0.69]	
Palestinian refugee camp	13 (9.2)	0.43 [0.05-0.77]	
Living status			
Live with family	135 (95.7)	0.32 [-0.02-0.67]	0.309
Live alone	6 (4.3)	0.23 [0.06-0.27]	
Education level			
No formal education	17 (12.1)	-0.03 [-0.16-0.22]	**0.026**
Primary	35(24.8)	0.41 [-0.04-0.72]	
Secondary	61 (43.3)	0.34 [ 0.07-0.62]	
Graduated	28(19.9)	0.42 [0.18-0.81]	
Marital status			
Single, Widow, Divorced	28(19.9)	0.14 [-0.09-0.27]	**0.001**
Married	113(80.1)	0.41 [0.04-0.71]	
Occupation			
Unemployed	126(89.4)	0.29 [-0.04-0.59]	**0.001**
Employed	15 (10.6)	0.68 [0.36-0.91]	
Household income (month)			
Low (less than 400 JD^a^)	71 (50.4)	0.28 [-0.04-0.62]	0.432
Moderate (400–1000 JD)	57 (40.4)	0.32 [0.06-0.71]	
High (more than 1000 JD)	13(9.2)	0.41 [0.18-0.87]	
Smoking			
Not smoker	109(77.3)	0.31 [-0.02-0.65]	0.976
Smoker	32 (22.7)	0.35 [-0.02-0.66]	
Diabetes duration			
1-3 years	8(5.7)	0.28 [-0.13-0.7]	0.829
4-5 years	3(2.1)	0.46 [0.04-]	
More than 5 years	130(92.2)	0.31 [-0.02-0.64]	
Dialysis vintage (Years)			
Less than 4 years	87 (61.7)	0.32 [-0.02-0.69]	0.835
4 years or more	54 (38.3)	0.31 [-0.03-0.62]	
Dialysis frequency			
2 days or less	18 (12.8)	0.14 [-0.18-0.54]	0.117
3 days or more	123 (87.2)	0.32 [0.02-0.66]	
Dialysis session duration (hours)			
Less than 4 hours	120 (85.1)	0.31 [-0.02-0.64]	0.901
4 hours or more	21 (14.9)	0.32 [-0.02-0.7]	
Kidney transplant			
Yes	3 (2.1)	0.46 [0.178-	0.466
No	138 (97.9)	0.31 [-0.02-0.64]	
Total chronic diseases			
None	7 (5)	0.77 [0.61-0.88]	**0.021**
One	48 (34)	0.31 [0.14-0.61]	
Two	46 (32.6)	0.36 [0.01-0.67]	
Three or more	40 (28.4)	0.11 [-0.09-0.51]	
Total chronic medications			
Less than 8	92 (65.2)	0.31 [-0.07-0.59]	0.301
8 or more	49 (34.8)	0.32 [0.07-0.70]	
HbA1c			
Controlled(<7)	92 (65.2)	0.29 [0.01-0.66]	0.596
Uncontrolled(>7)	49 (34.8)	0.41 [-0.05-0.65]	

*Abbreviations*: *BMI* body mass index, *JD* Jordanian Dinar, *HbA1c* glycated haemoglobin

^a^1 Jordanian Dinar (JD) equals 1.41 US Dollar

^b^The *p*-values are bold where they are less than the significance level cut-off of 0.05

Table 3 presents the multivariate associations between HRQOL scores and patients’ gender, education level, marital status, occupational status, and total chronic diseases. As shown in Table 3, marital status and occupational status were significantly (p < 0.05) associated with the QOL score. More specifically, married status and being employed were positively associated with QOL score (β = 0.22; *p* = 0.016 and β = 0.27; *p* = 0.013, respectively).

**Table 3:** Multivariate associations between health-related quality of life score^a^ and four predictors among diabetic patients undergoing hemodialysis

Variables^b, c^	Unstandardized Coefficients		Standardized Coefficients	t	*P* value^d^
	B	Std. Error	Beta		
Gender					
Male	Ref.				0.207
Female	-0.09	0.07	-0.12	-1.27	
Education level					
Under-university levels	Ref.				0.380
University level	0.07	0.08	0.07	0.88	
Marital status					
Single, Widow, Divorced	Ref.				**0.016**
Married	0.22	0.09	0.21	2.45	
Occupational status					
Unemployed	Ref.				**0.013**
Employed	0.27	0.11	0.21	2.51	
Total chronic diseases					
Less than three	Ref.				0.065
Three or more	-0.13	0.07	-0.15	-1.86	

^a^health-related quality of life is a five-item scale assessing five separate dimensions of health (high score meaning better quality of life)

^b^Univariate variables with *p* values < 0.05 were entered into the multiple linear regression

^c^Nominal variables were entered into analyses using dummy coding

^d^The *p*-values are bold where they are less than the significance level cut-off of 0.05

## Discussion

In our study, we have evaluated the HRQOL among hemodialysis patients with DM at the North of West Bank, Palestine. Although HRQOL among patients undergoing hemodialysis in Palestine was studied previously [19], and also HRQOL among Palestinian diabetic patients [6], there are no data regarding HRQOL specifically between diabetic patients undergoing hemodialysis. We evaluated the HRQOL using the EQ-5D. On reviewing the literature, HRQOL was assessed in diabetic patients using the EQ-5D in various nations [2, 6, 26–30]; also, this scale was used to measure HRQOL among patients undergoing hemodialysis [19, 31–33], but our study is the first of its kind to use the EQ-5D to assess HRQOL among diabetic dialysis patients. Diabetic dialysis patients have significantly more complications and lower HRQOL compared to diabetic patients not undergoing hemodialysis [5, 34].

Our study showed that the mean EQ-5D score in hemodialysis patients with DM was 0.314 (SD 0.4) with mean EQ-VAS score for same patients of 50.85 (SD 22.43). Because no studies used these scores among diabetic dialysis patients, we were unable to compare EQ-5D score and EQ-VAS score means with any from past research. However, other studies using the same instrument among diabetic patients found the mean EQ-5D score as follows: Palestine 0.7 ± 0.20 [6], Norway 0.81 (SD 0.22) [2]. Moreover, the mean EQ-5D score among patients on hemodialysis was 0.37 ± 0.44 in Palestine [19]. The mean EQ-5D score among diabetic dialysis patients in our study was lower than that of diabetic patients or patients on hemodialysis, which was expected due to the combination of complications resulting from both DM and hemodialysis. However, it was obvious that HRQOL in patients on hemodialysis alone or diabetic dialysis patients is worse than that of patients with DM alone, showing the significance of dialysis and its complications on patients’ quality of life, which is consistent with conclusions from many previous studies [11, 35].

This study has shown that female patients had lower HRQOL than male patients. Other studies on diabetic patients found the same result. For example, Wexler et al.’s study [36] exploring correlates of HRQOL in type 2 DM using the Health Utilities Index-III found that female sex was one of the significant factors associated with decreased patient HRQOL. Another study [19] on Palestinian patients on hemodialysis using EQ-5D score found the same result. This result can be explained by the nature of females’ lifestyle in a developing country such as Palestine, which is mainly a sedentary lifestyle [37], and the low level of involvement of females in social activities. Passchier et al. emphasizes the major consequences of social activities for patients’ QOL [38]. Moreover, patients who were single, divorced or widowed were found to have significantly decreased HRQOL. That is consistent with a previous study that found marital status to be a significant predictor of HRQOL among diabetic patients in Gaza [39]. Additional international studies found the same result [40, 41]. A possible explanation for this might be that patients who aren’t married are more likely to have poor family support and to be isolated socially, so this can lead to decreased adherence to the prescribed hemodialysis and other treatment orders [42].

Our results demonstrated that increased educational level was associated with higher HRQOL. This may be because patients with a higher education level are likely to have a better understanding of their illness, its complications, and the importance of adherence to dialysis sessions and other treatment modalities [43]. Many studies found that education level is a significant determinant of HRQOL among both diabetic patients [6, 43] and patients on hemodialysis [19, 38, 44]. Furthermore, we found that unemployed patients had lower HRQOL than employed patients. Javanbakht et al. [43] found in a study on Iranian diabetic patients that most patients who answered having extreme problems in the items on the EQ-5D were unemployed. Unemployment was also found to be highly associated with poor HRQOL scores in other studies on hemodialysis patients [19, 44–46]. This seems logical, as unemployed patients are more likely to be depressed [47], be socially inactive and have lower income, which can lead to impaired patients’ HRQOL [48].

As for clinical factors, we found that the more chronic diseases the patient had, the more likely he/she was to have poor HRQOL scores. A study on Iranian Muslim patients undergoing hemodialysis found that having DM or other comorbidity was related to patients’ QOL [49]. In addition, Yang et al. [50] studied HRQOL in Singapore and found that low Charlson comorbidity index was significantly associated with better HRQOL in Asian patients with renal failure. This finding can be explained as the more comorbidities the patient has, the lower physical activity and as a result QOL he will have [51].

### Strengths and limitations

Notwithstanding the relatively limited sample, this work had many positive points. For example, it was the first study of our knowledge to use EQ-5D-5L among diabetic patients undergoing hemodialysis. Moreover, this was the first study in Palestine to assess HRQOL in diabetic dialysis patients. In addition, we included patients from both urban and rural areas because of the multi-center design of the study. On the other hand, there were some limitations we have to mention. First of all, we can’t identify a cause-effect relationship because of the cross-sectional nature of the study. Secondly, we didn’t include a generalized sample, as we included only six dialysis centers out of 11 dialysis centers in West Bank. Thirdly, the absence of control groups (i.e. diabetic patients not on maintenance dialysis and non-diabetic patients on maintenance dialysis) limits the interpretation of the disease burden on HRQOL. Lastly, there were some clinical and social factors that might affect patients’ QOL that we didn’t include in our study, such as diet, and some laboratory values.

## Conclusions

Among diabetic patients undergoing hemodialysis, married status and being employed were associated with modestly higher QOL scores. In order to improve the QOL of diabetic patients undergoing hemodialysis, we recommend that healthcare providers give more attention to diabetic dialysis patients who are unemployed and unmarried, as they are at higher risk of having impaired HRQOL.

## Additional file

Additional file 1: Study questionnaires. This is the final version of the English and Arabic version that was used to obtain data which will help to address the factors associated with impaired health-related quality of life (HRQOL) in hemodialysis patients with diabetes in Palestine. (DOCX 107 kb)

## Abbreviations

BMI: Body mass index
DM: Diabetes mellitus
EQ-5D-5L: Five-level EuroQol five-dimensional instrument
EQ-VAS: Euroqol-visual analogue scale
ESRD: End-stage renal disease
HbA1c: Glycated haemoglobin
HRQOL: Health-related quality of life
IRB: INSTITUTIONAL Review Board
JD: Jordanian dinner
MOH: Ministry of Health
QOL: Quality of life
SD: Standard deviation

## References

[1] Roglic G, Unwin N (2010). Mortality attributable to diabetes: estimates for the year 2010. Diabetes Res Clin Pract.

[2] Solli O, Stavem K, Kristiansen IS (2010). Health-related quality of life in diabetes: The associations of complications with EQ-5D scores. Health Qual Life Outcomes.

[3] Di Lullo L, Mangano M, Ronco C, Barbera V, De Pascalis A, Bellasi A, Russo D, Di Iorio B, Cozzolino M (2017). The treatment of type 2 diabetes mellitus in patients with chronic kidney disease: What to expect from new oral hypoglycemic agents. Diabetes Metab Syndr.

[4] Caramori ML, Mauer M (2003). Diabetes and nephropathy. Curr Opin Nephrol Hypertens.

[5] Osthus TB, von der Lippe N, Ribu L, Rustoen T, Leivestad T, Dammen T, Os I (2012). Health-related quality of life and all-cause mortality in patients with diabetes on dialysis. BMC Nephrol.

[6] Zyoud SH, Al-Jabi SW, Sweileh WM, Arandi DA, Dabeek SA, Esawi HH, Atyeh RH, Abu-Ali HA, Sleet YI, Abd-Alfatah BM (2015). Relationship of treatment satisfaction to health-related quality of life among Palestinian patients with type 2 diabetes mellitus: Findings from a cross-sectional study. J Clin Transl Endocrinol.

[7] Fukuhara S, Yamazaki S, Hayashino Y, Green J (2007). Measuring health-related quality of life in patients with end-stage renal disease: why and how. Nat Clin Pract Nephrol.

[8] Dashti-Khavidaki S, Sharif Z, Khalili H, Badri S, Alimadadi A, Ahmadi F, Gatmiri M, Rahimzadeh S (2013). The use of pharmaceutical care to improve health-related quality of life in hemodialysis patients in Iran. Int J Clin Pharm.

[9] Jacobson AM, de Groot M, Samson JA (1994). The evaluation of two measures of quality of life in patients with type I and type II diabetes. Diabetes Care.

[10] Sorensen VR, Mathiesen ER, Watt T, Bjorner JB, Andersen MV, Feldt-Rasmussen B (2007). Diabetic patients treated with dialysis: complications and quality of life. Diabetologia.

[11] Fukuhara S, Lopes AA, Bragg-Gresham JL, Kurokawa K, Mapes DL, Akizawa T, Bommer J, Canaud BJ, Port FK, Held PJ (2003). Health-related quality of life among dialysis patients on three continents: the Dialysis Outcomes and Practice Patterns Study. Kidney Int.

[12] Landman GW, van Hateren KJ, Kleefstra N, Groenier KH, Gans RO, Bilo HJ (2010). Health-related quality of life and mortality in a general and elderly population of patients with type 2 diabetes (ZODIAC-18). Diabetes Care.

[13] Kleefstra N, Landman GW, Houweling ST, Ubink-Veltmaat LJ, Logtenberg SJ, Meyboom-de Jong B, Coyne JC, Groenier KH, Bilo HJ (2008). Prediction of mortality in type 2 diabetes from health-related quality of life (ZODIAC-4). Diabetes Care.

[14] Kalantar-Zadeh K, Kopple JD, Block G, Humphreys MH (2001). Association among SF36 quality of life measures and nutrition, hospitalization, and mortality in hemodialysis. J Am Soc Nephrol.

[15] Martinez-Castelao A, Gorriz JL, Garcia-Lopez F, Lopez-Revuelta K, De Alvaro F, Cruzado JM (2004). Perceived health-related quality of life and comorbidity in diabetic patients starting dialysis (CALVIDIA study). J Nephrol.

[16] Ministry of Health, Palestinian Health Information Center. Health Annual Report Palestine 2015. 2016 [cited; Available from: http://www.moh.ps/Content/Books/NWNJXX7RJ92Bn4f5EGYiH43a2tjAAzKBnseGnEUCaqWqYZndsbCcPy_JQWguvkHTR4Xk4zUpdT45ooWxH11BhIbVAxwpGWy2wiwHdGcM5K7aZ.pdf.

[17] Al-Jabi SW, Zyoud SH, Sweileh WM, Wildali AH, Saleem HM, Aysa HA, Badwan MA, Awang R (2015). Relationship of treatment satisfaction to health-related quality of life: findings from a cross-sectional survey among hypertensive patients in Palestine. Health Expect.

[18] Abu Farha NH, Khatib MT, Salameh H, Zyoud SH (2017). Cancer-related post-treatment pain and its impact on health-related quality of life in breast cancer patients: a cross sectional study in Palestine. Asia Pac Fam Med.

[19] Zyoud SH, Daraghmeh DN, Mezyed DO, Khdeir RL, Sawafta MN, Ayaseh NA, Tabeeb GH, Sweileh WM, Awang R, Al-Jabi SW (2016). Factors affecting quality of life in patients on haemodialysis: a cross-sectional study from Palestine. BMC Nephrol.

[20] Horowitz E, Abadi-Korek I, Shani M, Shemer J (2010). EQ-5D as a generic measure of health-related quality of life in Israel: reliability, validity and responsiveness. Isr Med Assoc J.

[21] Garcia-Alcala H, Ruiz-Arguelles A, Cedillo-Carvallo B (2009). Effect of the method to measure levels of glycated hemoglobin on individual clinical decisions: comparison of an immunoassay with high-performance liquid chromatography. Am J Clin Pathol.

[22] Sonthon P, Promthet S, Changsirikulchai S, Rangsin R, Thinkhamrop B, Rattanamongkolgul S, Hurst CP (2017). The impact of the quality of care and other factors on progression of chronic kidney disease in Thai patients with Type 2 Diabetes Mellitus: A nationwide cohort study. PLoS One.

[23] EuroQOL Group. EQ-5D-5L User Guide: Basic information on how to use the EQ-5D-5L instrument. 2011 [cited 2013 January 7]; Available from: http://www.euroqol.org/fileadmin/user_upload/Documenten/PDF/Folders_Flyers/UserGuide_EQ-5D-5L.pdf.

[24] Zyoud SH, Al-Jabi SW, Sweileh WM, Wildali AH, Saleem HM, Aysa HA, Badwan MA, Awang R, Morisky DE (2013). Health-related quality of life associated with treatment adherence in patients with hypertension: a cross-sectional study. Int J Cardiol.

[25] EuroQol Group. EQ-5D-5L Crosswalk Index Value Calculator. 2016 [cited 2016 November 30]; Available from: http://www.euroqol.org/fileadmin/user_upload/Documenten/Excel/Crosswalk_5L/EQ-5D-5L_Crosswalk_Index_Value_Calculator.v2.xls.

[26] Clarke P, Gray A, Holman R (2002). Estimating utility values for health states of type 2 diabetic patients using the EQ-5D (UKPDS 62). Med Decis Making.

[27] Lee WJ, Song KH, Noh JH, Choi YJ, Jo MW (2012). Health-related quality of life using the EuroQol 5D questionnaire in Korean patients with type 2 diabetes. J Korean Med Sci.

[28] Redekop WK, Koopmanschap MA, Stolk RP, Rutten GE, Wolffenbuttel BH, Niessen LW (2002). Health-related quality of life and treatment satisfaction in Dutch patients with type 2 diabetes. Diabetes Care.

[29] Sakamaki H, Ikeda S, Ikegami N, Uchigata Y, Iwamoto Y, Origasa H, Otani T, Otani Y (2006). Measurement of HRQL using EQ-5D in patients with type 2 diabetes mellitus in Japan. Value Health.

[30] Ragnarson Tennvall G, Apelqvist J (2000). Health-related quality of life in patients with diabetes mellitus and foot ulcers. J Diabetes Complications.

[31] Manns B, Johnson J, Taub K, Mortis G, Ghali W, Donaldson C (2003). Quality of life in patients treated with hemodialysis or peritoneal dialysis: what are the important determinants?. Clin Nephrol.

[32] Manns BJ, Johnson JA, Taub K, Mortis G, Ghali WA, Donaldson C (2002). Dialysis adequacy and health related quality of life in hemodialysis patients. ASAIO J.

[33] Tajima R, Kondo M, Kai H, Saito C, Okada M, Takahashi H, Doi M, Tsuruoka S, Yamagata K (2010). Measurement of health-related quality of life in patients with chronic kidney disease in Japan with EuroQol (EQ-5D). Clin Exp Nephrol.

[34] Ahola AJ, Saraheimo M, Forsblom C, Hietala K, Sintonen H, Groop PH (2010). Health-related quality of life in patients with type 1 diabetes--association with diabetic complications (the FinnDiane Study). Nephrol Dial Transplant.

[35] Merkus MP, Jager KJ, Dekker FW, de Haan RJ, Boeschoten EW, Krediet RT (2000). Predictors of poor outcome in chronic dialysis patients: The Netherlands Cooperative Study on the Adequacy of Dialysis. The NECOSAD Study Group. Am J Kidney Dis.

[36] Wexler DJ, Grant RW, Wittenberg E, Bosch JL, Cagliero E, Delahanty L, Blais MA, Meigs JB (2006). Correlates of health-related quality of life in type 2 diabetes. Diabetologia.

[37] Merom D, Sinnreich R, Aboudi V, Kark JD, Nassar H (2012). Lifestyle physical activity among urban Palestinians and Israelis: a cross-sectional comparison in the Palestinian-Israeli Jerusalem risk factor study. BMC Public Health.

[38] Kao TW, Lai MS, Tsai TJ, Jan CF, Chie WC, Chen WY (2009). Economic, social, and psychological factors associated with health-related quality of life of chronic hemodialysis patients in northern Taiwan: a multicenter study. Artif Organs.

[39] Eljedi A, Mikolajczyk RT, Kraemer A, Laaser U (2006). Health-related quality of life in diabetic patients and controls without diabetes in refugee camps in the Gaza strip: a cross-sectional study. BMC Public Health.

[40] Papadopoulos AA, Kontodimopoulos N, Frydas A, Ikonomakis E, Niakas D (2007). Predictors of health-related quality of life in type II diabetic patients in Greece. BMC Public Health.

[41] Trief PM, Wade MJ, Britton KD, Weinstock RS (2002). A prospective analysis of marital relationship factors and quality of life in diabetes. Diabetes Care.

[42] Untas A, Thumma J, Rascle N, Rayner H, Mapes D, Lopes AA, Fukuhara S, Akizawa T, Morgenstern H, Robinson BM (2011). The associations of social support and other psychosocial factors with mortality and quality of life in the dialysis outcomes and practice patterns study. Clin J Am Soc Nephrol.

[43] Javanbakht M, Abolhasani F, Mashayekhi A, Baradaran HR, Jahangiri noudeh Y (2012). Health related quality of life in patients with type 2 diabetes mellitus in Iran: a national survey. PLoS One.

[44] Lopes AA, Bragg-Gresham JL, Goodkin DA, Fukuhara S, Mapes DL, Young EW, Gillespie BW, Akizawa T, Greenwood RN, Andreucci VE (2007). Factors associated with health-related quality of life among hemodialysis patients in the DOPPS. Quality of Life Research.

[45] Al Wakeel J, Al Harbi A, Bayoumi M, Al-Suwaida K, Al Ghonaim M, Mishkiry A (2012). Quality of life in hemodialysis and peritoneal dialysis patients in Saudi Arabia. Ann Saudi Med.

[46] Garcia-Llana H, Remor E, Selgas R (2013). Adherence to treatment, emotional state and quality of life in patients with end-stage renal disease undergoing dialysis. Psicothema.

[47] Stankunas M, Kalediene R, Starkuviene S, Kapustinskiene V (2006). Duration of unemployment and depression: a cross-sectional survey in Lithuania. BMC Public Health.

[48] Park HC, Yoon HB, Son MJ, Jung ES, Joo KW, Chin HJ, Oh KH, Lim CS, Kim YS, Ahn C (2010). Depression and health-related quality of life in maintenance hemodialysis patients. Clin Nephrol.

[49] Saffari M, Pakpour AH, Naderi MK, Koenig HG, Baldacchino DR, Piper CN (2013). Spiritual coping, religiosity and quality of life: a study on Muslim patients undergoing haemodialysis. Nephrology (Carlton).

[50] Yang F, Griva K, Lau T, Vathsala A, Lee E, Ng HJ, Mooppil N, Foo M, Newman SP, Chia KS (2015). Health-related quality of life of Asian patients with end-stage renal disease (ESRD) in Singapore. Qual Life Res.

[51] O'Shea MP, Teeling M, Bennett K (2015). Comorbidity, health-related quality of life and self-care in type 2 diabetes: a cross-sectional study in an outpatient population. Ir J Med Sci.

