# Supplementary material for: Quality of life in hemodialysis diabetic patients: a multicenter cross-sectional study from Palestine
Source: BMC Nephrol. 2018 Feb 28;19:49. doi: 10.1186/s12882-018-0849-x (PMC5831192; doi:10.1186/s12882-018-0849-x)
Supplement: Supplementary file 1 — Study questionnaires. This is the final version of the English and Arabic version that was used to obtain data which will help to address the factors associated with impaired health-related quality of life (HRQOL) in hemodialysis patients with diabetes in Palestine. (DOCX 107 kb) [file 12882_2018_849_MOESM1_ESM.docx]

**Additional file 1.** **Study questionnaires. This is the final version of the** **English and Arabic version that was used to obtain data which will help to address the factors associated with impaired health-related quality of life (HRQOL) in hemodialysis patients with diabetes in Palestine**

**English version**

**Section 1**

................... 1-Age

2-Gender ❑Male ❑Female

3-Height ................... 4-Weight..................

5- Residency ❑Palestinian refugee camp ❑village ❑city

6- Living status ❑live alone ❑live with family

7- Education level ❑no formal education ❑ primary ❑secondary ❑Graduated

8- Marital status ❑single, widow, divorced ❑ married

9- Occupation ❑Employed ❑Unemployed

10- Household income ❑ (month) Low (less than 400 JD) ❑Moderate (400–1000 JD) ❑High (more than 1000 JD)

11-Are you a smoker ❑ Yes ❑No

**Section 2**

12-How many years you are on regular haemodialysis? ........................

13-How many years you are having Diabetes Mellitus? ........................

14-What is the number of dialysis sessions per week?

❑ 2 days or less ❑ 3 days ❑4 days ❑5days ❑ more than 5

15-How much time each dialysis session last? ......................

16-Have you ever had Kidney Transplantation?

❑Yes ❑ No

17-Do you have other diseases?

❑Hypertension

❑Arthritis

❑Systemic Lupus Erythromatosus

❑Coronary artery disease

❑Stroke

❑Heart Failure

❑Lung disease

Others................................................

18-What are your regular medications

1-............................... 2-.......................................... 3. ......................................

4- ....................... 5- .......................................... 6- .....................................

Others ....................................

19-Do you have your medications by yourself?

❑Yes ❑ No

20- patient’s HbA1c ......................................

**EQ-5D-5L**

Under each heading, please tick the ONE box that best describes your health TODAY

**MOBILITY**

I have no problems in walking about ❑

I have slight problems in walking about ❑

I have moderate problems in walking about ❑

I have severe problems in walking about ❑

I am unable to walk about ❑

**SELF-CARE**

I have no problems washing or dressing myself ❑

I have slight problems washing or dressing myself ❑

I have moderate problems washing or dressing myself ❑

I have severe problems washing or dressing myself ❑

I am unable to wash or dress myself ❑

**USUAL ACTIVITIES** *(e.g. work, study, housework,*

*family or leisure activities)*

I have no problems doing my usual activities ❑

I have slight problems doing my usual activities ❑

I have moderate problems doing my usual activities ❑

I have severe problems doing my usual activities ❑

I am unable to do my usual activities ❑

**PAIN / DISCOMFORT**

I have no pain or discomfort ❑

I have slight pain or discomfort ❑

I have moderate pain or discomfort ❑

I have severe pain or discomfort ❑

I have extreme pain or discomfort ❑

**ANXIETY / DEPRESSION**

I am not anxious or depressed ❑

I am slightly anxious or depressed ❑

I am moderately anxious or depressed ❑

I am severely anxious or depressed ❑

I am extremely anxious or depressed ❑

- We would like to know how good or bad your health is

10

0

20

30

40

50

60

80

70

90

100

5

15

25

35

45

55

75

65

85

95

The best health
 you can imagine

The worst health
 you can imagine

TODAY.

- This scale is numbered from 0 to 100.
- 100 means the best health you can imagine.
  0 means the worst health you can imagine.
- Mark an X on the scale to indicate how your health is TODAY.
- Now, please write the number you marked on the scale in the box below.

YOUR HEALTH TODAY =

**Arabic version**

**عزيزي المشارك، إن الغرض من هذه الاستمارة هو تقييم جودة الحياة عند المرضى الذين يعانون من فشل كلوي ومرض السكري ويخضعون لغسيل الكلى، لذا نرجو منك الإجابة عن الأسئلة التالية، علما بأن المعلومات التي ستدلي بها ستظل سرية وتستخدم فقط لأغراض البحث العلمي، وشكرا لكم مقدما.**

**ملاحظة: الاستمارة مكتوبة في صيغة المذكر إلا أنها موجهة لكلا الجنسين على حد سواء**

**تتكون هذه الاستمارة من خمسة أقسام:**

**القسم الأول:**

**1-العمر:**...................

**2- الجنس:** ❑ ذكر ❑ انثى

**3- الطول:**  ..................**4 - الوزن** .......................................

**5- مكان الإقامة :** ❑ مخيم ❑ قرية ❑ مدينة

**6- إقامتك الحالية :**❑ أعيش وحدي ❑ أعيش مع عائلتي ❑ غير ذلك ..............................

**7-ما المستوى التعليمي لديك** :❑ غير دارس ❑ابتدائي ❑ إعدادي ❑ثانوية عامة

❑ كلية ( دبلوم ) ❑ جامعي ( بكالوريوس) ❑ دراسات عليا

**8- الحالة الاجتماعية:**❑ أعزب ❑متزوج ❑ مطلق ❑ أرمل

**9- ما نوع عملك؟**❑لا أعمل ❑ موظف ❑ غير موظف ❑ ربة منزل

**10- كم يبلغ معدل الدخل الشهري للعائلة؟**

❑أقل من 2000 شيكل❑ 2000-5000 شيكل❑ 5000-10000 شيكل

❑ أكثر من 10000شيكل

**11- هل أنت مدخن**؟ ❑ نعم ❑ لا

=============================================================

**القسم الثاني:**

**12- كم من الوقت مر على بداية عملية غسيل الكلى .........................................**

**13- كم من الوقت مر منذ تم تشخيصك بالسكري ............................**

**14**- **كم يوماً بالأسبوع تخضع لعملية غسيل الكلى:**

❑يومان أو أقل ❑ ثلاثة أيام

❑ أربعة أيام ❑ خمسة أيام ❑ أكثر من ذلك، حدد ................................

**15- كم ساعة تستغرق عملية الغسيل في كل مرة** ............................

**16**- **هل سبق وقمت بعملية زراعة الكلى:**❑ نعم ❑لا

**17- هل لديك أمراض أخرى**❑ سكري ❑ضغط الدم ❑ التهاب المفاصل

❑الذئبة الحمراء الجهازية ❑ ذبحة صدرية ❑جلطة دماغية.............................❑قصور في عضلة القلب❑ مرض رئوي مزمن

❑غير ذلك، اذكرها...........................................................................

**18**- **ما هي الأدوية التي تتناولها**

1-..............................................2-................................................3-......................................

4- ........................................... 5-............................................6-........................................

غير ذلك .....................................

**19- هل تتناول دواءك بمفردك؟❑** نعم ❑ لا

**20-Hb1AC(فحص السكر التراكمي): ..................................**

| ضع علامة في المربع الخاص بكل مجموعة في الأسفل، لتشير إلى أفضل عبارة تصف حالتك الصحية اليوم. | | |
| --- | --- | --- |
|  | القدرة على **التنقل** | |
| ❑ | **ليس لدي أي مشاكل عند المشي** | |
| ❑ | أعاني من مشاكل طفيفة **عند** المشي | |
| ❑ | **أعاني من مشاكل متوسطة عند المشي** | |
| ❑ | أعاني من مشاكل حادة **عند** المشي | |
| ❑ | ليس لدي القدرة على المشي | |
|  | **قدرتي على الاهتمام بنفسي** |  |
| ❑ | **ليس لدي**أي مشاكل في الاستحمام أوارتداء ملابسي بنفسي |  |
| ❑ | أعاني من مشاكل طفيفة في الاستحمام أوارتداء الملابس بنفسي |  |
| ❑ | **أعاني من مشاكل متوسطة** عند الاستحمام أو ارتداء الملابس بنفسي |  |
| ❑ | أعاني من مشاكل حادة عند الاستحمام أو ارتداء الملابس بنفسي |  |
| ❑ | ليس لدي القدرة على الاستحمام أو ارتداء الملابس بنفسي |  |
|  | الأنشطة المعتادة***(مثل العمل، الدراسة، الأعمال المنزلية***،*النشاطات* ***الأسرية أو الترفيهية)*** |  |
| ❑ | **ليس لدي** أي مشاكل في ممارسة نشاطاتي المعتادة |  |
| ❑ | أعاني من مشاكل طفيفة في القيام بنشاطاتي المعتادة |  |
| ❑ | **أعاني من مشاكل متوسطة في ممارسة نشاطاتي المعتادة** |  |
| ❑ | أعاني من مشاكل حادة في ممارسة نشاطاتي المعتادة |  |
| ❑ | ليس لديّ القدرة على ممارسة نشاطاتي المعتادة |  |
|  | الألم/الإحساس بعدم الراحة |  |
| ❑ | ليس لدي أي ألم أو إنزعاج |  |
| ❑ | أعاني من ألم طفيف أو إنزعاج طفيف |  |
| ❑ | أعاني من ألم متوسط أو إنزعاج متوسط |  |
| ❑ | أعاني من ألم حاد أو إنزعاج حاد |  |
| ❑ | أعاني من ألم شديد جداً أو إنزعاج شديد جداً |  |
|  | **قلق / اكتئاب** |  |
| ❑ | لا أعاني من أي قلق أو اكتئاب |  |
| ❑ | أعاني من قلق طفيف أو اكتئاب طفيف |  |
| ❑ | أعاني من قلق متوسط أو إكتئاب متوسط |  |
| ❑ | أعاني من قلق حاد أو اكتئاب حاد |  |
| ❑ | أعاني من قلقاً شديداً جداً أو اكتئاباً شديداً جداً |  |

*Israel (Arabic)© 2010 EuroQol Group EQ-5D™ is a trade mark of the EuroQol Group*

| - نود أن نعرف مدى جودة أو سوء صحتك **اليوم**. |
| --- |
| - هذا المقياس مدرج من الرقم 0 حتى 100. - الرقم 100 يعني أحسن حالة صحية يمكنك تصوّرها. 0 يعني **أسوأ** حالة صحية يمكنك تصوّرها. |
| - ضع X على المقياس للإشارة إلى وضعك الصحي **اليوم**. |
| - الآن، قم رجاء بكتابة الرقم الذي أشرت إليه على المقياس في الصندوق أدناه. |

أفضل وضع صحّي يُمكن تصوّره

**حالتك الصحية اليوم**=

أسوأ وضع صحّي يُمكن تصوره

0

5

10

15

20

25

30

35

40

45

50

55

60

65

70

75

80

85

90

95

100

*Israel (Arabic)© 2010 EuroQol Group EQ-5D™ is a trade mark of the EuroQol Group*
